# Large language model assisted decision support framework for uncertainty aware detection and management of tomato lateral shoots

**DOI:** 10.3389/fpls.2026.1853269

**Published:** 2026-06-02

**Authors:** Yingchun Jiang, Yanbin Geng, Zedong Zheng, Xinfu Pang

**Affiliations:** 1 College of Engineering, Shenyang Agricultural University, Shenyang, China; 2 School of Computing and Mathematical Sciences, University of Leicester, Leicester, United Kingdom; 3 School of Automation, Shenyang Institute of Engineering, Shenyang, China

**Keywords:** CBAM, LLM, tomato lateral shoots detection, uncertainty analysis, YOLOv8n-seg

## Abstract

Accurate identification of tomato lateral shoots is essential for automated pruning and plant monitoring in greenhouse production. However, complex illumination, leaf occlusion, and morphological variability often reduce detection reliability in optical vision systems. This study proposes an optical vision-based framework that integrates deep learning perception with large language model assisted pruning decision support. A tomato lateral Shoot image dataset was constructed using RGB imaging in greenhouse environments. A lightweight YOLOv8n instance segmentation model with the Convolutional Block Attention Module (CBAM) was developed to enhance feature representation. Data augmentation strategies were applied to simulate illumination variations and improve model robustness. Model interpretability was analyzed using Principal Component Analysis (PCA) and Gradient weighted Class Activation Mapping (Grad CAM). Experimental results show that the proposed YOLOv8n-seg+CBAM model achieves a mAP_0.5_ of 98.1% with only 3.28M parameters and an average inference time of 8.0 ms per image. Monte Carlo Dropout was further introduced to estimate the spatial uncertainty of cutting points. These structured perception features were provided to a large language model (LLM), enabling context aware pruning decision assistance. The proposed framework integrates vision-based shoot detection, uncertainty estimation, and LLM-assisted reasoning into a unified pipeline, enabling more reliable pruning decisions and improving safety and robustness compared with vision-only approaches in greenhouse environments.

## 1 Introduction

Tomatoes are widely cultivated worldwide as a high-value crop. Each leaf axil, where the main stem meets the leaf, contains a potential axillary bud that can develop into a lateral shoot under favorable conditions. If not removed promptly, these shoots can use up 30%−40% of photosynthetic products, which hinders fruit growth, lowers sugar content (Andres and Koskela, 2022), and may cause flower and fruit drop. Excessive lateral shoot growth results in dense plant canopies, shading leaves, and reduced airflow and light penetration. In greenhouse settings, increased air humidity promotes the spread of diseases like gray mold, leaf mold, and late blight (Zhang et al., 2021). Managing lateral shoot growth through pruning during growth stages is essential to ensure good tomato yield and quality. Traditional manual pruning is not only inefficient but also difficult to scale up due to labor shortages in agriculture. With China’s annual tomato production reaching 65 million tons—making up 35% of the world’s supply—automated pruning has become an urgent need for smart farming.

**Figure 1:**
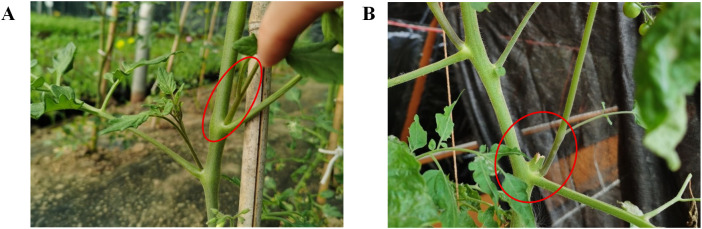
Image of tomato lateral shoots. **(A)** Growth status. **(B)** Regeneration status.

Tomato lateral shoots show strong branching and grow quickly. Regardless of the plant’s height, any shoot emerging from the main stem should be removed when it exceeds about 5 cm, and this task should be performed regularly to promote continuous main stem growth. **Figure 1A** illustrates the growth pattern of tomato lateral shoots. Timing of pruning is crucial. Pruning too early can hinder root development while delaying it may affect fruit growth and lower yield. Tomato lateral shoots also possess notable regenerative capacity. If the growth point at the leaf axil is not fully removed, remaining cells may continue dividing after cutting the shoot, resulting in regrowth, as shown in **Figure 1B**. Therefore, image recognition is essential for developing automated tomato pruning systems. It must identify multiple lateral shoot targets within complex backgrounds and provide accurate position information to robotic manipulators to prevent missed pruning or incorrect identification of the main stem. These requirements demand high accuracy and fast inference from the model.

### 1.1 Literature review

In recent years, with the ongoing progress in computer vision and deep learning, object detection and instance segmentation methods based on convolutional neural networks have been more frequently used in modern agriculture. These techniques provide vital visual information for tasks such as fruit and vegetable harvesting, precision pesticide application, and detection in complex agricultural settings.

#### 1.1.1 Multi-task learning in fruit and vegetable harvesting

In the field of fruit and vegetable harvesting, multi-task learning supports both object recognition and ripeness assessment (Wang T. et al., 2023) proposed a Mask-RCNN-based approach for locating tea picking points (Nan et al., 2023) introduced the WGB-YOLO model for dragon fruit detection in dense orchards (Li et al., 2023c) developed a lightweight YOLOv4-based method for tea bud detection (Huang et al., 2025) presented an improved YOLOv8-based model for citrus ripeness detection (He et al., 2025b) applied YOLOv8n-seg for broccoli recognition and harvesting posture estimation, and (He et al., 2025a) further combined YOLOv8s with YOLOv5-cls for strawberry harvestability classification. However, these studies mainly focus on single-task optimization or independent perception tasks, and the integration of multi-task learning with unified decision-making for harvesting operations remains limited.

#### 1.1.2 Precision pesticide application

In precision pesticide application, deep learning models have become the main method for pest and disease recognition (Peng et al., 2024) proposed PestNet based on MobileNet-V2 for lightweight pest classification (Dai et al., 2024) introduced an improved YOLOv8-GABNet for citrus pest detection (Chen S. Y. et al., 2024) proposed an image enhancement-based method combining denoising and filtering for corn seed crack detection. (Kunduracioglu and Pacal, 2024) applied fine-tuned CNNs for grape pest classification. Although these methods improve classification accuracy and efficiency, most of them focus on local region recognition, while fine-grained spatial localization and downstream decision support remain insufficiently explored.

#### 1.1.3 Complex agricultural environments

In agricultural environments, background interference such as support structures and reflective films significantly affects detection performance (Liu et al., 2024) proposed MAE-YOLOv8 for green plum detection (Wang X. F. et al., 2023) developed a lightweight YOLOv5-based tomato picking detection model (Meng et al., 2024) introduced attention mechanisms into YOLOv7-Tiny for small and occluded tomato detection (Li et al., 2024) developed an improved YOLOv5-based mulberry branch recognition model. Despite these improvements, current methods still struggle with strong occlusion, dense canopies, and illumination variations, indicating limited robustness in real-world field conditions.

#### 1.1.4 Tomato pruning point detection

In tomato pruning, existing research mainly focuses on identifying lateral shoots and pruning points (Feng et al., 2022) developed a Mask R-CNN-based pruning point recognition method (Liang et al., 2022) improved Mask R-CNN using MobileNetV3-Large and ECA attention for lightweight detection (Liang et al., 2025) proposed an improved YOLOv5-based pruning model achieving 92.0% accuracy (Liang and Wei, 2025) further combined CycleGAN with YOLOv8 for nighttime segmentation, reaching 93.3% accuracy. However, most existing approaches focus on detection or segmentation alone, while the transformation from perception results to reliable pruning decision-making is still insufficient, especially under complex and low-illumination conditions.

#### 1.1.5 Large language models in agricultural decision support

Recent studies have begun to explore data-driven reasoning and intelligent decision support in agricultural systems (Owais et al., 2026) proposed a SAMConvFormer framework that integrates visual perception models with large language models for crop detection and management recommendation (Zhao et al., 2024) introduced an LLM-based crop growth modeling method within a digital twin platform to simulate multi-stage crop development (Liu et al., 2026) proposed a time-aware retrieval-augmented generation framework for pest and disease management, incorporating temporal knowledge to improve decision quality. These studies indicate an increasing research focus on integrating advanced reasoning models into agricultural decision-making systems. However, current approaches are still loosely coupled with visual perception modules, and the direct linkage between real-time detection results and decision-level reasoning remains limited, which motivates further exploration of integrated perception and decision frameworks.

A summary of related work is presented in **Supplementary Table 1**.

### 1.2 Research gap and contributions

The studies reviewed above indicate that computer vision and deep learning–based image recognition techniques have been extensively applied in the agricultural domain. In tasks such as fruit and vegetable identification, ripeness assessment, and pest and disease monitoring, researchers have obtained reliable recognition performance by developing convolutional neural networks, object detection models, and image segmentation frameworks. Deep learning methods have shown practical feasibility when addressing recognition problems involving small targets in complex agricultural environments. Similarly, the identification of tomato lateral shoots can be approached using these methodologies.

Although existing image recognition models can detect drooping tomato leaves in factory cultivation systems (**Supplementary Figure 1**), these systems generally adopt single stem pruning. Where only the main vine is kept and all other branches and leaves are removed (Yang et al., 2024). Unlike relatively independent targets with clear boundaries, such as fruits and leaves, identifying tomato lateral shoots that develop naturally in greenhouse environments, as shown in **Supplementary Figure 2**, is more complex (Li et al., 2023b). First, lateral shoots emerge from axillary points on the main stem and are often tangled with the stem and surrounding leaves, creating complex spatial arrangements. Second, the color and texture of lateral shoots closely resemble those of nearby stems and foliage, making traditional edge- or color-based segmentation methods less effective. Additionally, variable lighting conditions in greenhouses or open fields, along with shadows, background interference, and overlapping foliage, often cause missed detections and false positives. Therefore, accurately and efficiently detecting tomato lateral shoots in greenhouse environments remains a challenge that needs further research.

Deep learning-based instance segmentation models can enhance the accuracy of lateral Shoot detection. However, under conditions of complex occlusion and similar features, the model outputs may still have some level of uncertainty. If automatic pruning decisions rely only on visual model predictions, the chance of incorrect pruning could rise, potentially impacting plant growth quality and system safety. Therefore, relying solely on low-level visual features is not enough to meet the reliability standards for automated pruning in greenhouses environments.

To address the issues discussed above, the main contributions of this study are as follows:

1. A tomato lateral shoot dataset was collected and annotated under greenhouse cultivation conditions. The samples cover diverse lighting conditions, shooting angles, and growth stages, reflecting the complexity of representative greenhouse conditions. During training, multiple data augmentation strategies, including brightness adjustment, color perturbation, rotation, and flipping, were applied to simulate environmental variations. These strategies improved the model’s adaptability to illumination changes and morphological differences.
2. Under identical data conditions, Mask R-CNN, YOLOv8, and YOLOv11 were compared using mAP, inference speed, and parameter count. Based on the results, the lightweight YOLOv8 segmentation model was selected as the baseline. Three attention mechanisms, SE, ECA, and CBAM, were incorporated separately for evaluation. The results show that CBAM improves channel and spatial feature fusion. The model demonstrates stronger focus on lateral shoots and main stems. Feature representation capability is improved accordingly.
3. To address fluctuations in cutting point localization caused by leaf occlusion and structural ambiguity in greenhouse environments, Monte Carlo Dropout was applied to perform multiple stochastic predictions of cutting points. A spatial distribution model was constructed to quantify localization uncertainty. Based on these results, constraint conditions were defined using cutting point uncertainty, lateral shoot length, and occlusion ratio. An LLM was further introduced for semantic analysis of multidimensional features. This process supports auxiliary decision-making for pruning and improves system stability and reliability under complex conditions.
4. PCA was used for feature dimensionality reduction and visualization. Grad-CAM was applied to analyze weight distribution. These methods illustrate the model’s learning characteristics related to the structural features of tomato lateral shoots. The results help explain recognition behavior in complex plant backgrounds and provide a reference for interpretability analysis in agricultural vision models.

The structure of the other parts of this study is as follows: The second section details the specific steps for identifying tomato lateral shoots and the data collection method; the third section describes the image annotation process and the model structure; the fourth section presents the simulation experiments and discusses their interpretability; the fifth section provides the conclusion.

## 2 Problem description

This study addresses the problem of accurate identification and safe pruning decisions for tomato lateral shoots in complex greenhouse environments. An integrated framework was developed that combines visual perception, uncertainty assessment, and semantic reasoning. Tomato plant images were collected under different growth stages, lighting conditions, and background disturbances to construct an annotated dataset. This dataset improves adaptability to practical agricultural scenarios. To address challenges such as slender shoot morphology, similarity to main stems and leaves, and frequent occlusion, a deep segmentation network was designed with multi-scale feature enhancement and attention mechanism optimization. The network improves localization performance of lateral shoots. On this basis, an uncertainty quantification method was introduced to assess the stability of pruning point predictions. An LLM was further incorporated to provide auxiliary semantic reasoning for pruning decisions. The proposed framework enhances decision reliability while maintaining recognition performance. This study provides visual and intelligent decision support for automated tomato pruning and lateral shoot management in greenhouse environments.

**Figure 2:**
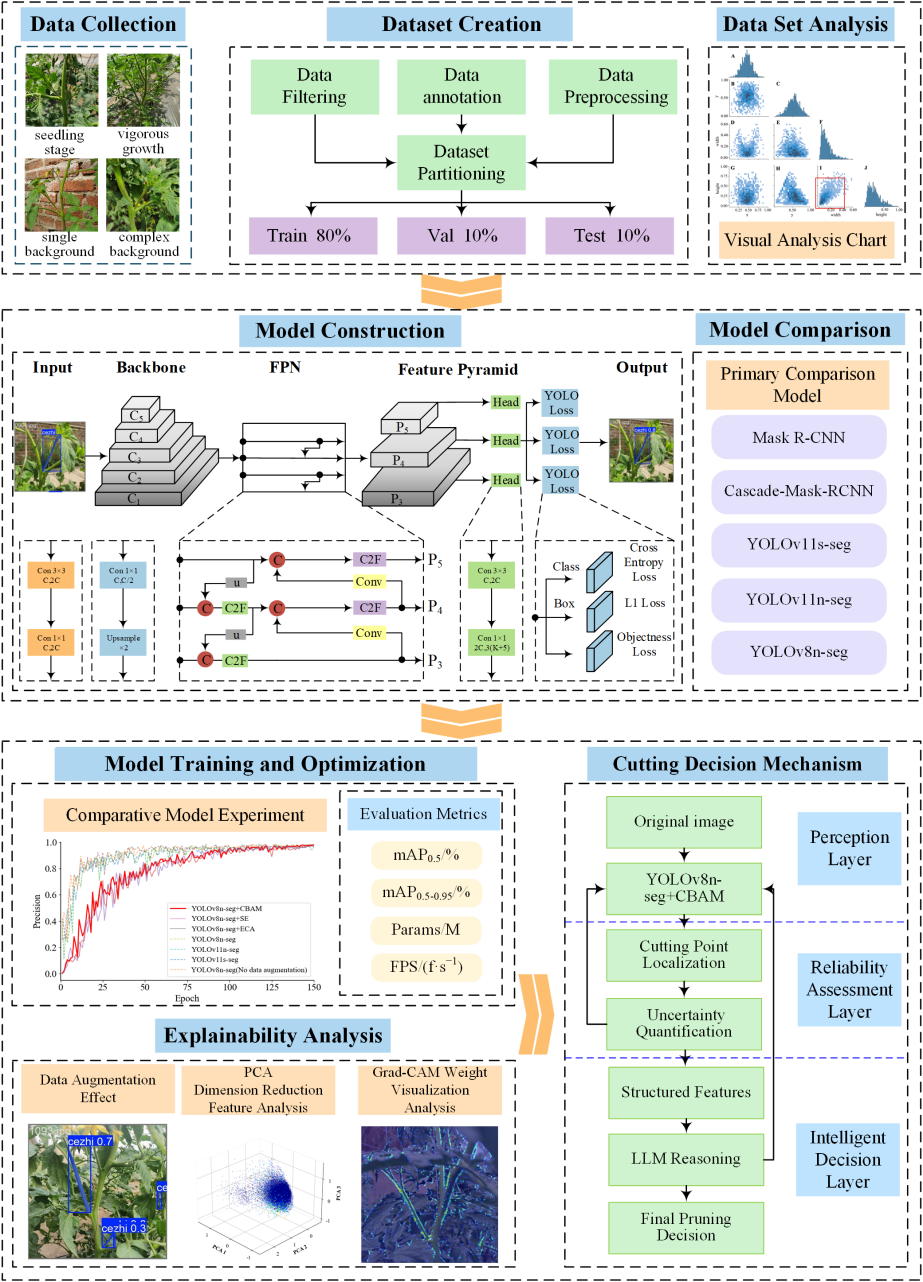
Tomato lateral shoots identification strategy diagram.

**Figure 2** illustrates the tomato lateral shoot identification strategy. The overall structure of the proposed method can be divided into three steps:

### 2.1 Stage 1: data collection and preprocessing

To recognize tomato lateral shoots, data collection and preprocessing were initially carried out. In a greenhouse, high-definition cameras captured numerous images of tomato plants, including lateral shoots at various growth stages, under different lighting conditions, and from multiple angles. Pixel-level annotation of the Tomato lateral shoots was then performed using the LabelMe segmentation tool. After annotation, the dataset was proportionally divided to support model training and enhance robustness.

### 2.2 Stage 2: model construction

The model consists of four components: the Backbone, which extracts features from tomato shoots and leaves; the Neck, which fuses features across different scales; the Head, which outputs mask regions for tomato lateral shoots; and a composite loss function, which allows the model to perform detection and segmentation of tomato lateral shoots.

### 2.3 Stage 3: model training and optimization

Using the preprocessed dataset, the Mask R-CNN series, YOLOv8 series, and YOLOv11 series models were trained and evaluated. The decline curves of the loss function and the accuracy metrics on the validation set were recorded, and the accuracy of the predicted bounding boxes and segmentation masks was comprehensively assessed on the test set. Model optimization was carried out by incorporating attention mechanisms, applying data augmentation, and adjusting learning rates, optimizers, and regularization parameters. Interpretability analysis was conducted using two methods: PCA feature visualization for dimensionality reduction and Grad-CAM weight visualization to examine the model’s focus on Tomato lateral shoots.

### 2.4 Stage 4: uncertainty assessment of pruning points and LLM assisted decision making

Based on the lateral shoot segmentation results, candidate pruning points are determined according to the spatial relationship between lateral shoots and the main stem. During inference, Monte Carlo Dropout is applied to perform multiple stochastic forward passes. The mean and variance of the predicted cutting points are calculated to quantify localization uncertainty. Constraint conditions are then defined using lateral shoot length, occlusion ratio, and uncertainty level. These structural features and uncertainty metrics are provided to an LLM for semantic analysis. The model generates auxiliary pruning decisions and corresponding confidence scores.

## 3 Data preprocessing

### 3.1 Data acquisition

**Figure 3:**
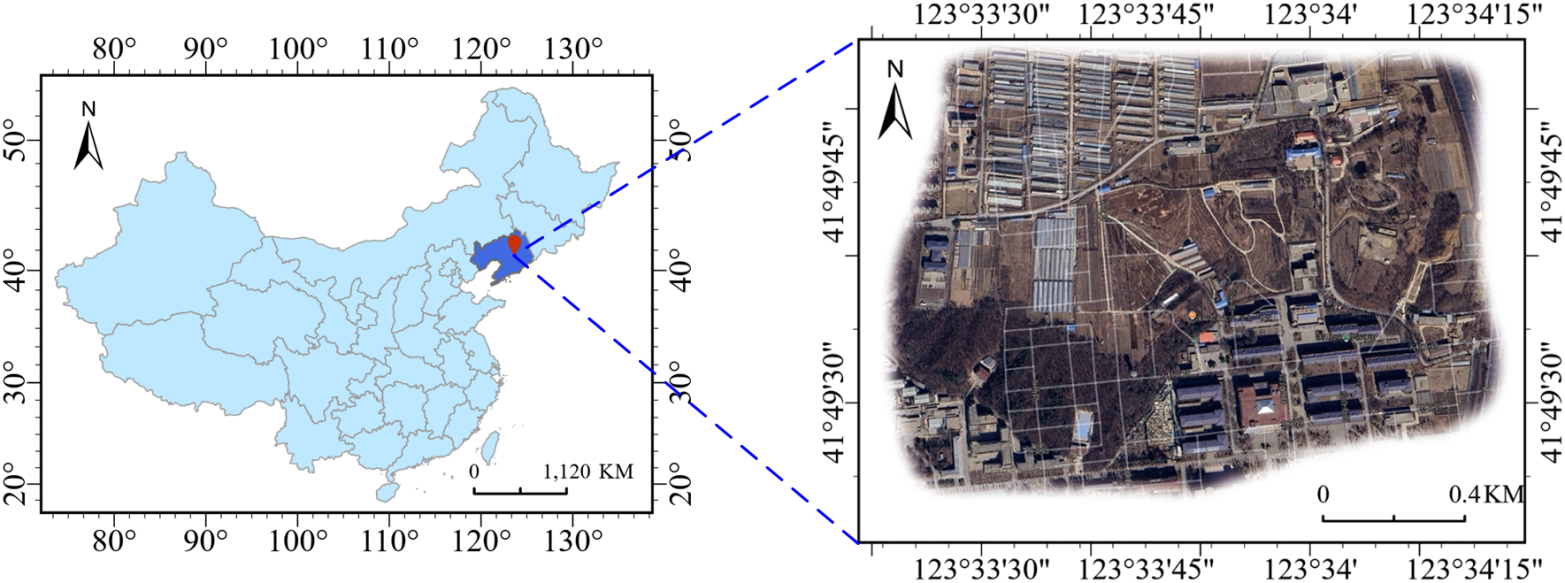
Dataset shooting location.

**Figure 4:**
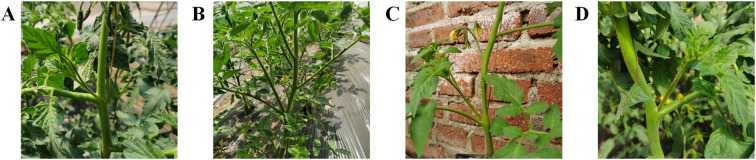
The initial image of tomato lateral shoots. **(A)** Seedling stage. **(B)** Vigorous growth stage. **(C)** Simple background. **(D)** Complex background.

Image data were collected in Greenhouses 38 and 39 at the Shenyang Agricultural University Greenhouse Base (**Figure 3**), located in Shenyang, Liaoning Province, China, at 41°49′N and 123°33′E, with an elevation of approximately 40−50 m. Images were taken during two time periods,9:00-11:00 and 16:00-18:00. The camera operated in automatic exposure mode, with a shooting distance of 300 to 450 mm. A total of 1,130 images were captured across different growth stages and viewing angles. Each image had a resolution of 2736 × 2736 pixels (1:1 aspect ratio) and was saved in JPG format. **Figure 4** shows representative raw images of tomato lateral shoots under various morphological and background conditions, covering four categories: seedling stage, vigorous growth stage, single background, and complex background.

### 3.2 Dataset construction and annotation

The LabelMe tool was used for manual mask annotation, where precise pixel-level contours were drawn for each Tomato lateral shoot to ensure complete boundary coverage. In areas with overlap or occlusion, annotation priority was given to maintaining the segment ability of the lateral shoots to reduce sample noise. All masks were consistently assigned the category label “cezhi,” and the annotation results were exported in COCO format. This annotation approach is suitable for instance segmentation tasks, as it provides more detailed spatial information than bounding boxes and supports more accurate detection in complex backgrounds. After annotation, the dataset was divided into training, validation, and test sets in an 8:1:1 ratio. The training set was used for parameter learning, the validation set for performance evaluation and hyperparameter tuning during training, and the test set for an independent assessment of the final model to minimize overfitting.

**Figure 5:**
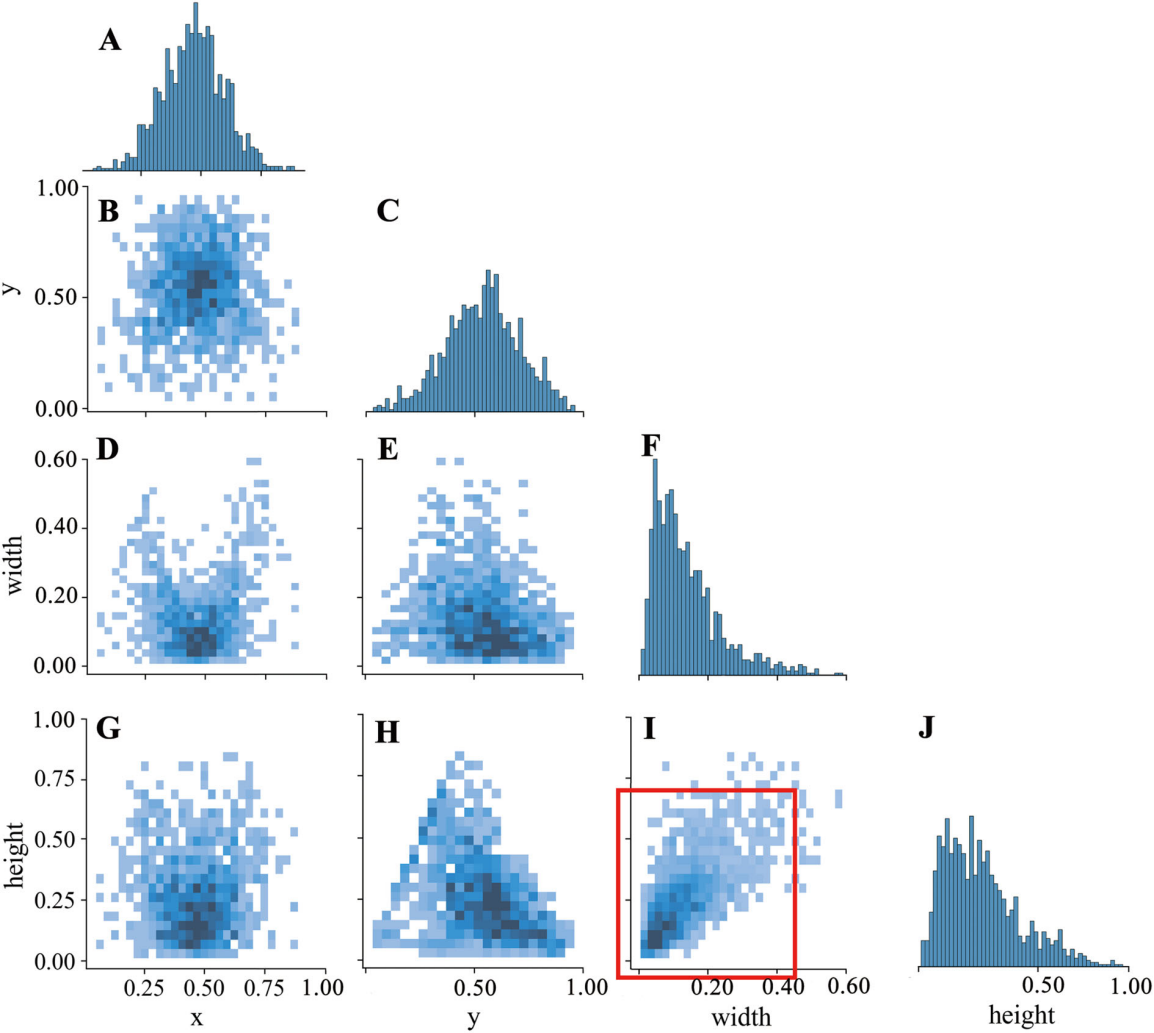
Data set visualization analysis. **(A)** Horizontal position distribution of targets. **(B)** Spatial distribution of targets in the image. **(C)** Vertical position distribution of targets. **(D)** Relationship between target width and horizontal position. **(E)** Relationship between target width and vertical position. **(F)** Distribution of target widths. **(G)** Relationship between target height and horizontal position. **(H)** Relationship between target height and vertical position. **(I)** Relationship between target height and width. **(J)** Distribution of target heights.

To assess the dataset’s validity, **Figure 5** offers a visualization analysis of the annotated masks. Statistical evaluations were conducted on pixel area, shape, and positional distribution. Targets with an aspect ratio of less than 0.1 relative to the image were classified as small objects (Li et al., 2018). As shown by the red box in **Figure 5I**, small Tomato lateral shoot masks made up a large proportion, and their area distribution showed a long-tail pattern. The masks’ positional distribution was evenly spread across the images, showing that various parts of the plant were covered during data collection. Therefore, when creating the model, it’s important to focus on detailed information of small targets and to increase the input resolution of the original image.

As shown in the univariate distributions in **Figures 5A, C, F, J**, the locations of Tomato lateral shoot targets are mainly focused in the central area of the images. This indicates that the shooting angle during data collection was quite consistent, with minimal variation in the relative positions of the camera and the plants. The scatter matrices in **Figures 5B, D, E, G, H, I** illustrate the relationships between variables. A positive correlation is seen between width and height, suggesting that most targets have elongated shapes that align with the growth traits of Tomato lateral shoots.

However, as shown by the distribution of center coordinates in **Figure 5B**, the samples are concentrated within a relatively small spatial area, indicating that the range of shooting angles is limited. This clustered distribution can easily cause overfitting to specific environments or angles during model training, which can hurt the model’s ability to generalize in complex environments. To improve the model’s robustness against different lighting conditions, perspective changes, and background complexity, it is important to include data augmentation strategies during training. This artificially creates diverse training data to boost the model’s generalization abilities.

### 3.3 Data augmentation

To enhance the model’s generalization ability and performance, as well as to enable it to adapt to different scenarios and variations, transformations such as rotation, translation, and scaling are applied to the training data. These operations help reduce overfitting and increase sample diversity, thereby improving accuracy and robustness in real-world object detection tasks (Upadhyay et al., 2025; Nagaraju et al., 2022). In this study, a set of data augmentation techniques is used. By adding controlled perturbations in both color and geometric domains, the model is better equipped to learn the characteristic variations of tomato lateral shoots across different scenarios during training. The specific augmentation strategies are summarized in **Supplementary Table 2**.

The augmentation strategies described above are carried out using the data augmentation module in the Ultralytics YOLOv8 framework. The effects of these augmentations are shown in **Supplementary Figure 3**. During each training iteration, the module randomly chooses augmentation operations and applies them to input images in real time, eliminating the need for offline preprocessing and preserving sample diversity. Some augmentation operations activate only during training and are automatically disabled during validation and testing to ensure stable and consistent inference results.

### 3.4 Overview of the YOLOv8 architecture and its main components

**Figure 6:**
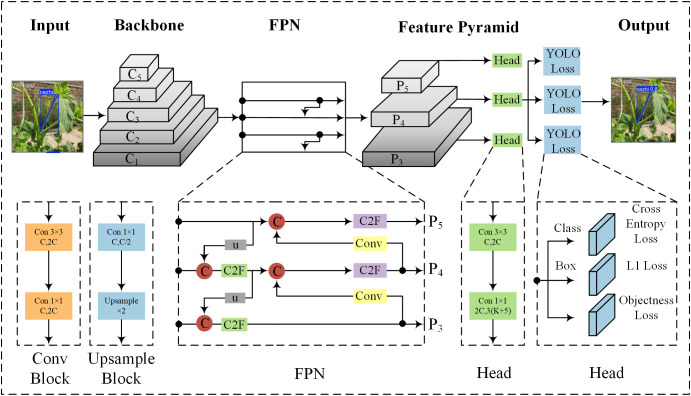
YOLOv8 architecture diagram.

YOLOv8 supports object detection, instance segmentation, and pose estimation. By integrating the C2f module, decoupled heads, and an anchor-free mechanism, it achieves a balanced improvement in accuracy and inference speed. In agricultural production, it offers clear advantages for plant growth monitoring, pest and disease identification, fruit detection, and harvesting. The YOLOv8-seg model extends the practicality and accuracy of the YOLO series by adding an instance segmentation branch, enabling pixel-level segmentation. Its overall architecture includes four components: a backbone network, multi-scale feature fusion modules (FPN and PAN), anchor-free heads for detection and segmentation, and a composite loss function, as shown in **Figure 6**. In this study, the model was trained using tomato plant images collected by the visual system to generate bounding boxes and masks for accurate segmentation and identification of tomato lateral shoots. This provides a technical foundation for the efficient operation of automated pruning machinery.

#### 3.4.1 Backbone

The core network of YOLOv8 uses the C2f module, which employs the Cross Stage Partial (CSP) (Wang et al., 2020; Zhang et al., 2022) design to improve gradient flow and cut down on redundant computations. This setup effectively captures fine details of leaves, fruits, and branches in tomato plant images, building a strong feature base for later detection tasks.

#### 3.4.2 FPN (feature pyramid network)

FPN is a multi-scale feature fusion network architecture that improves feature maps through a top-down approach. During fusion, lateral shoots maintain high-resolution details in shallow-layer features, ensuring thorough object detection across various scales. This boosts small object detection capabilities (Guo et al., 2023) and prevents information loss in lateral shoots during feature extraction.

#### 3.4.3 PAN (path aggregation network)

PAN builds upon FPN to further improve feature propagation efficiency and information flow, highlighting bottom-up information feedback. By adding bottom-up paths to FPN’s top-down architecture, it achieves more comprehensive feature fusion through bidirectional path aggregation, thereby enhancing detection capabilities for small objects and targets with complex boundaries.

YOLOv8 uses the multi-scale fusion PAN-FPN bidirectional path (Li et al., 2023a; Chen T. C. et al., 2024), enabling two-way transmission of high-level semantic information and low-level detail information to boost multi-scale object detection. When detecting tomato lateral shoots of different sizes, it shows superior sensitivity and localization accuracy.

#### 3.4.4 Head

The YOLOv8 head uses an anchor-free detection and segmentation mechanism with separate classification and regression shoots. The anchor-free design allows flexible adaptation to object scale variation without predefined anchor boxes. This design makes bounding box prediction simpler, decreases the need for extensive hyperparameter tuning, and directly predicts object center, size, and category, which enhances detection accuracy and convergence speed (Kong et al., 2020). When detecting Tomato lateral shoots, variations in plant shape and the complexity of lighting and backgrounds require the model to adapt to different object sizes and shapes. In these situations, using Anchor Boxes remains effective for capturing such variations.

#### 3.4.5 Loss function

YOLOv8 uses a complex combined loss function that includes bounding box loss, classification loss, focus loss, and mask loss for the YOLOv8-seg model employed in instance segmentation tasks. The overall loss function is defined as Equation 1:

(1) $$L(\theta )=\lambda_{box}\sum_{pos}L_{box}(\theta )+\lambda_{dlf}\sum_{pos}L_{cls}(\theta )+\lambda_{dfl}\sum_{pos}L_{dfl}(\theta )+\lambda_{seg}L_{seg}(\theta )+\frac{\phi }{2}{\left\|\theta \right\|}^{2}$$

Among these, *L*(*θ*) is the overall loss function used to optimize model parameters *θ*. *λ* is the weighting coefficient controlling the importance of this term; *L_box_*(*θ*) is the bounding box regression loss, measuring the discrepancy between predicted and ground-truth boxes; *L_cls_*(*θ*) is the classification loss, used for category classification to determine whether the predicted target category is correct; *L_dfl_*(*θ*) is the distributed focus loss, used to optimize the discrete coordinate distribution of bounding boxes and improve localization accuracy; *L_seg_*(*θ*) is the segmentation loss, optimizing the consistency between predicted and ground-truth masks. It comprises *L_BCE_*(*θ*) and *L_Dice_*(*θ*): *L_BCE_*(*θ*) is a per-pixel binary cross-entropy loss ensuring pixel-level classification accuracy, while *L_Dice_*(*θ*) measures the overlap between predicted and ground-truth masks to enhance segmentation of foreground regions. $\frac{\phi }{2}{\left\|\theta \right\|}^{2}$ is the regularization term, preventing overfitting and constraining the model’s parameter scale.

### 3.5 Attention mechanism

**Figure 7:**
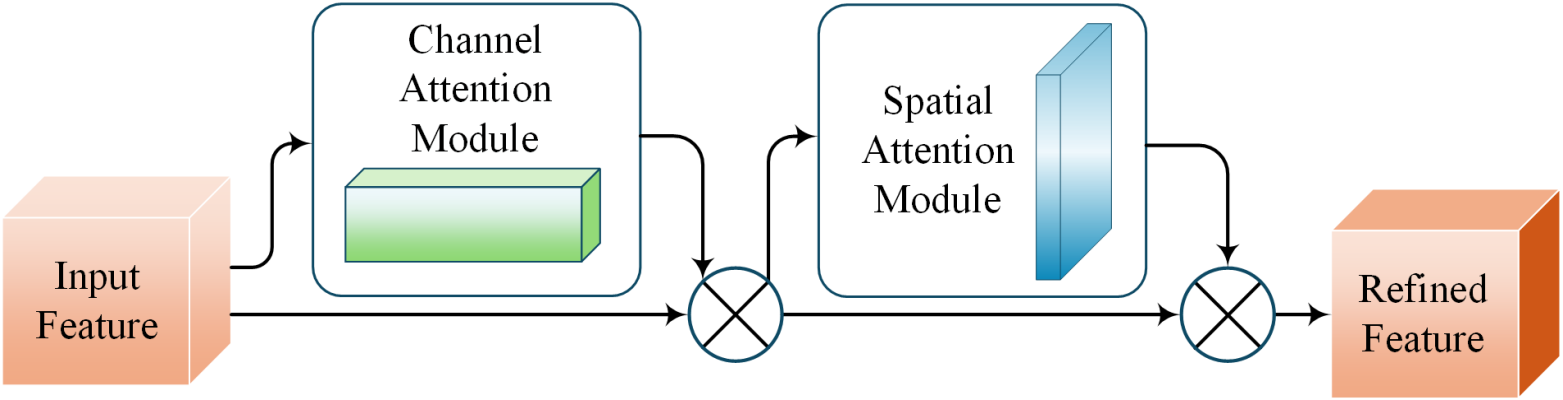
Dual-channel attention mechanism architecture.

When faced with scenes that have complex backgrounds and small targets, convolutional neural networks often distribute attention evenly across all feature regions, resulting in poor extraction of key features. Attention mechanisms, however, can imitate human visual focus by automatically adjusting weights. This enables the model to focus more on task-relevant areas while ignoring irrelevant information (Liu et al., 2023; Wang et al., 2022). Its structure is shown in **Figure 7**.

The Channel Attention Module identifies the feature channels that contribute most to the task, allowing the model to enhance its response to texture features at the edges and nodes of tomato stems while suppressing irrelevant features such as background leaves and soil. The Spatial Attention Module pinpoints the most important spatial regions, enabling the network to focus on areas where the main stem and tomato lateral shoots intersect and to reduce background interference. Embedding these two modules in series within the middle layers of the backbone improves edge information and local contrast. during feature extraction.

## 4 Experimental verification and evaluation

### 4.1 Experimental environment

Model training is conducted using the PyTorch deep learning framework in a high-performance GPU environment. Its API allows flexible customization of various model architectures. The Albumentations library is used to create a unified data augmentation pipeline, including operations like random rotation, color dithering, and multi-scale cropping. This method helps prevent training bias from affecting the model’s ability to generalize at the data source level. The training process employs MMDetection and Ultralytics for comprehensive monitoring, including dynamic loss function tuning, adaptive learning rate adjustments, and COCO metric validation. This systematic approach to model management ensures all models are trained fairly under the same protocols. **Supplementary Table 3** details the hardware and software configurations used in the experiments, establishing a baseline for reproducibility.

### 4.2 Model evaluation and recognition performance analysis

#### 4.2.1 Model evaluation metrics

In the Tomato lateral shoots identification task, lateral shoots are considered positive samples, while non-lateral shoots are considered negative samples. For targets predicted as “cezhi,” the precision (*P*) reflects the proportion of predictions that are true Tomato lateral shoots and serves as an indicator of the false positive rate. The formulation is shown in Equation 2:

(2) $$P=\frac{TP}{TP+FP}$$

where TP (True Positive) refers to the number of actual targets correctly identified as positive, while FP (False Positive) indicates the number of non-lateral shoot targets incorrectly classified as positive. A higher P-value suggests a lower likelihood of misclassifying leaves, fruits, and other structures as positive in complex background conditions.

Recall (*R*) measures the model’s ability to detect true Tomato lateral shoots and is calculated as shown in Equation 3:

(3) $$R=\frac{TP}{TP+FN}$$

where FN (False Negative) represents the number of positive samples that exist but are not detected. A higher *R* indicates fewer missed Tomato lateral shoots and supports a more complete extraction of effective structural information from tomato plants.

To further evaluate the model’s overall detection performance across various thresholds, this study uses Mean Average Precision (mAP) as the comprehensive performance metric, defined in Equations 5, 6: 

(4) $$AP=\int_{0}^{1}P(R)dR$$

(5) $$mAP=\frac{1}{N}\sum_{i=1}^{N}AP_{i}$$

where *P*(*R*) denotes the precision-recall curve with recall(*R*) on the x-axis. The focus is on the mAP performance at IoU thresholds of 0.5 and 0.5-0.95. mAP_0.5_ indicates the model’s detection accuracy at a single threshold, while mAP_0.5-0.95_ better illustrates the model’s overall stability across multiple scales and confidence levels.

#### 4.2.2 Visual analytics

**Figure 8:**
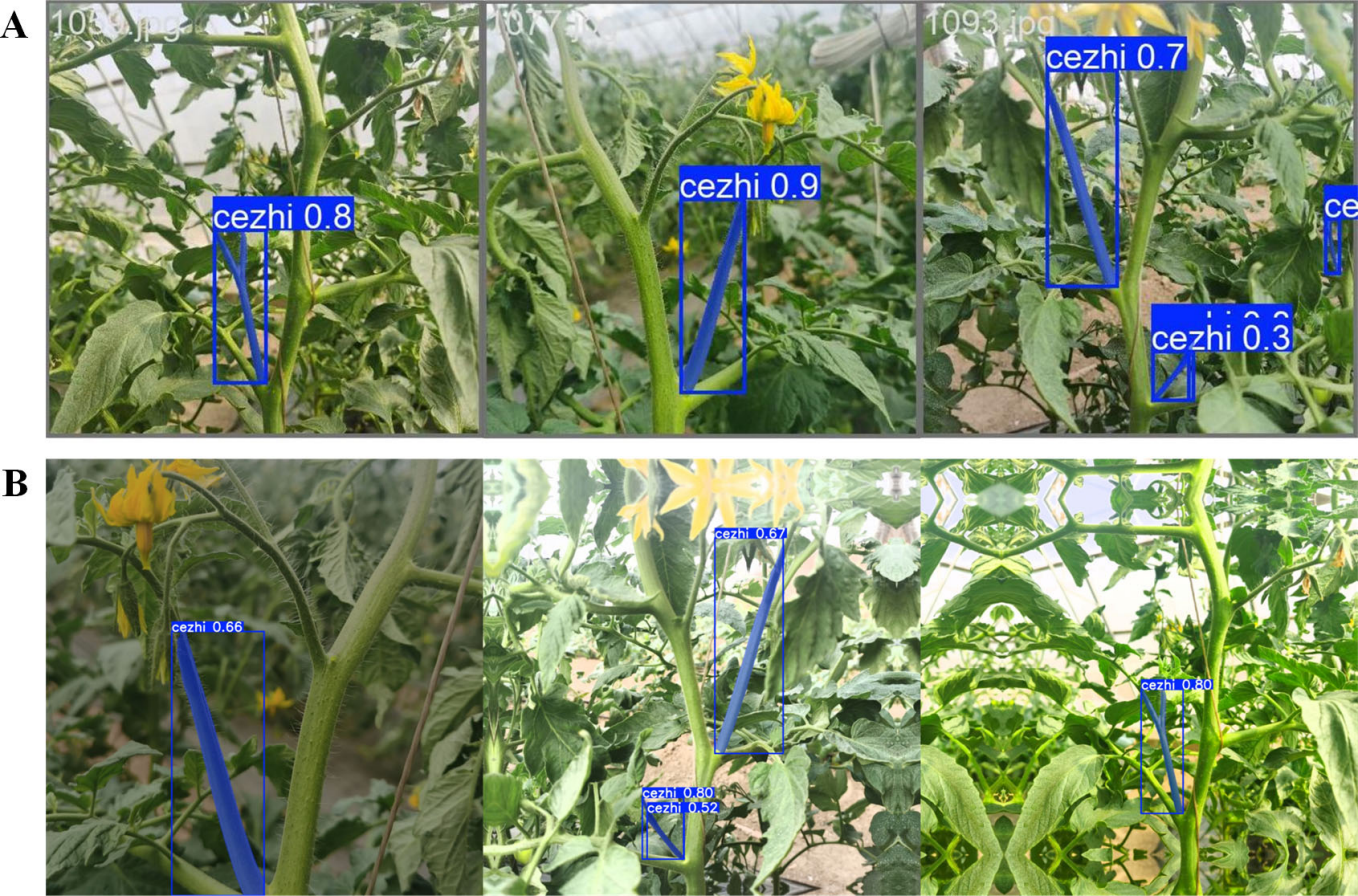
Example of data augmentation effect. **(A)** Training effect without data augmentation. **(B)** Training effect under data augmentation Training effect under data augmentation.

This study uses multiple data augmentation strategies listed in **Supplementary Table 2** during training to simulate tomato plant features under different lighting conditions in greenhouse environments. These methods help the model adapt to brightness changes caused by variations in light intensity, enhancing recognition stability under various image capture conditions. To assess the effectiveness of the lighting augmentation strategy, comparisons were made using models trained with and without augmentation. **Figure 8** shows examples of the augmentation effects. **Figure 8A** displays the training results without data augmentation, while **Figure 8B** presents results achieved with augmentation to mimic diverse lighting conditions. The recognition results demonstrate that the model trained with augmentation provides more accurate segmentation of tomato lateral shoots in difficult settings, including shadowed and reflective areas, and successfully reduces false positives and negatives.

### 4.3 Model performance comparison experiment

To validate the performance of the proposed model in tomato lateral shoot detection and segmentation tasks, this study conducted comparative experiments using leading two-stage detection models (Mask R-CNN, Cascade-Mask-RCNN) and one-stage detection models (YOLOv8n-seg, YOLOv11 series). For the main lightweight segmentation models, each experiment was independently repeated three times under the same training configuration, and the results are reported as mean ± standard deviation. **Table 1** shows the experimental results comparing average precision, parameter count, and speed across all models.

**Table 1:** Model comparison results.

Model	mAP_0.5_/%	mAP_0.5-0.95_/%	Params/M	FPS/(f·s^−1^)
Mask R-CNN	95.7	48.9	42	16.1
Cascade-Mask-RCNN	94.2	47.6	55	11.2
YOLOv11s-seg	97.5 ± 0.3	54.2 ± 0.6	10.1	38
YOLOv11n-seg	96.7 ± 0.3	54.1 ± 0.6	2.83	50
YOLOv8n-seg	97.4 ± 0.2	54.6 ± 0.1	3.3	101.1
YOLOv8n-seg(No data augmentation)	97.2	54.8	3.26	127.3

Analysis of the data shows that compared to Mask R-CNN and Cascade-Mask-RCNN, the YOLOv8n-seg model improves in mAP_0.5_ by 1.9% and 3.4%, respectively, and in mAP_0.5-0.95_ by 5.7% and 7%, respectively. The YOLOv8n-seg model exhibits higher accuracy than mainstream two-stage detection models. Despite this, the model’s parameter count (Params) is only 3.3M—down by 92.1% and 94% respectively—while inference speed (FPS) rises to 101.1 frames per second, showing significant lightweight and practical benefits. Although the YOLOv11 series models marginally improve accuracy, YOLOv11n-seg’s parameter count increases about three times, and its inference speed decreases by half.

Analysis of the data shows that compared to Mask R-CNN and Cascade-Mask-RCNN, the YOLOv8n-seg model improves in mAP0.5 by 1.7% and 3.2%, respectively, and in mAP0.5-0.95 by 5.7% and 7.0%, respectively. The YOLOv8n-seg model exhibits higher accuracy than mainstream two-stage detection models. Despite this, the model’s parameter count (Params) is only 3.3M—down by 92.1% and 94.0%, respectively—while inference speed (FPS) rises to 101.1 frames per second, showing significant lightweight and practical benefits. The experimental results indicate that YOLOv11s-seg achieves the highest segmentation accuracy (mAP_0.5_ = 97.5%), but its parameter count reaches 10.1M. In contrast, YOLOv11n-seg reduces the parameter size to 2.83M, although the segmentation accuracy decreases to 96.7%. Compared with the YOLOv11 series, YOLOv8n-seg achieves comparable segmentation accuracy (mAP_0.5_ = 97.4%) while maintaining a relatively small model size an, demonstrating better suitability for lightweight and real-time greenhouse deployment.

The experimental results indicate that YOLOv11s-seg achieves the highest segmentation accuracy (mAP_0.5_ = 97.5%), but its parameter count reaches 10.1M. In contrast, YOLOv11n-seg reduces the parameter size to 2.83M, although the segmentation accuracy decreases to 96.7%. Compared with the YOLOv11 series, YOLOv8n-seg achieves comparable segmentation accuracy (mAP_0.5_ = 97.4%) while maintaining a relatively small model size an, demonstrating better suitability for lightweight and real-time greenhouse deployment.

The YOLOv8n-seg model achieves very high inference speed and a small model size while maintaining high detection accuracy, making it highly suitable for embedded and real-time detection scenarios. **Supplementary Figure 4** shows the training curves for this model. **Supplementary Figure 4A** displays the training set loss curve, where both classification and segmentation losses continuously decrease and stabilize, indicating that the model has effectively learned the features of tomato lateral shoots. **Supplementary Figure 4B** shows the validation set loss curve. The segmentation loss gradually converges between 80 and 150 epochs without significant fluctuations, demonstrating strong model generalization capabilities. **Supplementary Figure 4C** presents the model accuracy curve, recording two evaluation metrics: mAP_0.5_ and mAP_0.5-0.95_. The model reaches peak performance during training, but the weights used for final evaluation and deployment are not directly taken from these peak moments. Instead, they are chosen based on strategies like early stopping to select a model version with better stability and generalization. **Supplementary Figure 4D** shows the learning rate curve, where the learning rate decreases linearly from an initial value of 0.0020 to 0 according to the plan, guiding the model to converge stably on the tomato lateral Shoot recognition task.

### 4.4 Ablation studies of different attention mechanisms

Although YOLOv8n-seg has shown good convergence and stable detection accuracy in this study, issues such as insufficient local feature extraction and inadequate attention to critical regions still occur in complex environments. To better improve the model’s ability to detect important features in challenging scenarios and reduce missed detections of lateral shoots, three attention mechanisms—SE, ECA, and CBAM—were added to the YOLOv8n-seg model. By adaptively assigning feature weights across both channel and spatial dimensions, the model can better focus on key parts of tomato plants, improving detection and segmentation accuracy. Experimental results are shown in **Table 2**.

**Table 2:** Attention mechanism experiment results.

Model	mAP_0.5_/%	mAP_0.5-0.95_/%	Params/M	FPS/(f·s^−1^)
YOLOv8n-seg	97.4 ± 0.2	54.6 ± 0.1	3.3	101.1
YOLOv8n-seg+SE	97.1	52.4	3.26	119
YOLOv8n-seg+ECA	97.2	51.1	3.24	119
YOLOv8n-seg+CBAM	97.7 ± 0.4	52.4 ± 0.9	3.28	117

After introducing the attention mechanism, the SE and ECA modules perform feature weighting across channels, improving the model’s ability to distinguish subtle differences between tomato main stems and lateral shoots, making feature extraction more selective. However, since neither module considers spatial positional information and the texture differences between tomato lateral shoots and main stems are relatively minor, the model’s segmentation accuracy slightly decreased, with mAP_0.5_ values of 97.1% and 97.2%, respectively. Nonetheless, inference speed increased to 119f·s^−1^, demonstrating that the lightweight attention structure helps boost the model’s real-time performance.

**Figure 9:**
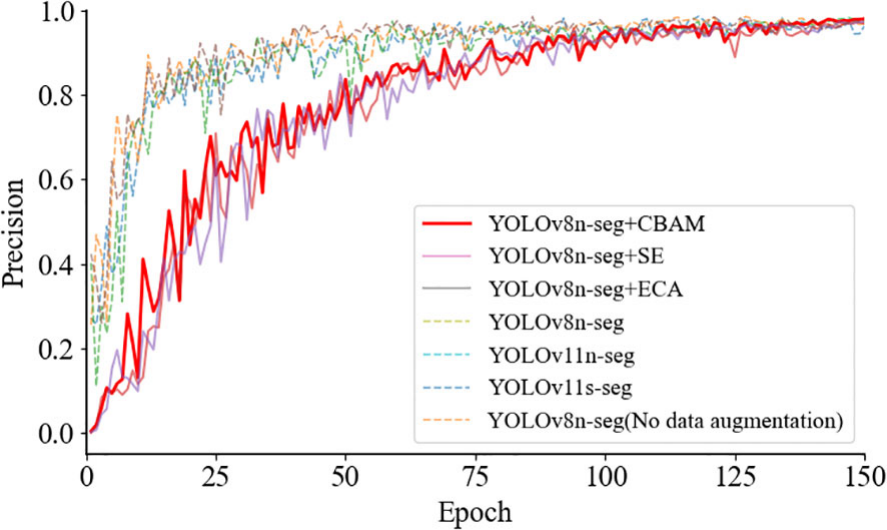
Recognition accuracy curves for each model.

In contrast, the CBAM module simultaneously combines channel attention and spatial attention. During feature extraction, it not only boosts responses from important channels but also guides the network to focus on key regions of tomato plants. This method enhances the model’s recognition ability under complex lighting and overlapping foliage. mAP_0.5_ increased to 97.7%, with lower rates of missed detections and false positives for lateral shoots. The inference speed reached 117f·s^−1^, processing 117 images per second, with an average inference time of about 8.0ms per image. **Figure 9** shows the convergence of the model’s accuracy curve under consistent data conditions.

The CBAM curve shows quick accuracy gains between 0 and 50 epochs, then steadily converges from 100 to 150 epochs. The SE and ECA curves have slightly lower accuracy and slower convergence compared to CBAM. Models without attention mechanisms exhibit notable oscillations during training, indicating limited generalization. Based on the curve profiles, the CBAM attention mechanism effectively assigns weights to channels and spatial features within feature maps. This quickly reduces background noise early in training, emphasizes important textures, clarifies gradient directions, and results in smoother convergence curves—all without adding to the model size.

### 4.5 Explainability analysis

To deepen the understanding of how features are learned and decisions are made in the tomato lateral shoot recognition task after adding the attention mechanism into the model, interpretability analysis was performed at both the data feature level and the model decision level. Using two visualization techniques, PCA-based dimensionality reduction for feature visualization and Grad-CAM weight visualization (Vellido, 2020), we examine the model’s ability to group features and its prediction reasoning. This analysis not only highlights behavioral patterns during feature extraction but also confirms the model’s effectiveness in identifying key features after integrating the attention mechanism.

#### 4.5.1 Feature visualization analysis using PCA for dimension reduction

In high-dimensional feature spaces, it is often difficult to intuitively visualize the differences between various tomato lateral shoot samples. Principal Component Analysis (PCA) is a data dimensionality reduction technique that condenses correlated high-dimensional variables into linearly independent low-dimensional variables. By reducing the dimensionality of the model’s intermediate layer features, they are mapped onto the first three principal component dimensions (PCA1, PCA2, PCA3) for three-dimensional visualization. **Supplementary Figure 5** shows a scatter plot visualization of the extracted tomato lateral shoot features. PCA analysis was applied to the feature vectors extracted from the two models, which were then projected into a three-dimensional space.

As shown in **Supplementary Figure 5A**, the feature points extracted by the original YOLOv8n-seg model are scattered across the three principal component spaces. They display significant variations in color and position and lack clear clustering patterns. There is substantial overlap among sample points, indicating the model’s limited ability to differentiate lateral shoots during feature extraction. This mixing of features suggests the model is affected by background noise when learning structural differences between shoots. As a result, the boundaries in the feature space become blurred, reducing discrimination accuracy.

In contrast, after introducing the CBAM attention mechanism, the model shows clear clustering patterns in the feature space, as illustrated in **Supplementary Figure 5B**. Sample points mainly cluster within specific regions, with significantly increased point cloud density, tighter grouping, and improved directionality. Feature points form distinct concentration trends along principal components PCA1 and PCA2, while showing hierarchical variation along PCA3. This indicates the model’s ability to capture branch structural changes at multiple scales. The PCA analysis results prove that adding the CBAM attention mechanism improves the feature extraction ability of the YOLOv8n-seg model. It produces structural and clustered distribution patterns in the low-dimensional feature space, reflecting the model’s accurate capture of key structural features in tomato lateral shoots.

#### 4.5.2 Visual analysis based on Grad-CAM weights

To further validate the impact of attention mechanisms on feature extraction for tomato lateral shoot recognition, we used Grad-CAM weight visualization analysis to examine the model’s feature responses. By calculating the gradient-weighted average of convolutional feature maps for target categories, we created heatmaps that show the model’s attention focus, visually revealing the image regions emphasized during prediction.

**Figure 10:**
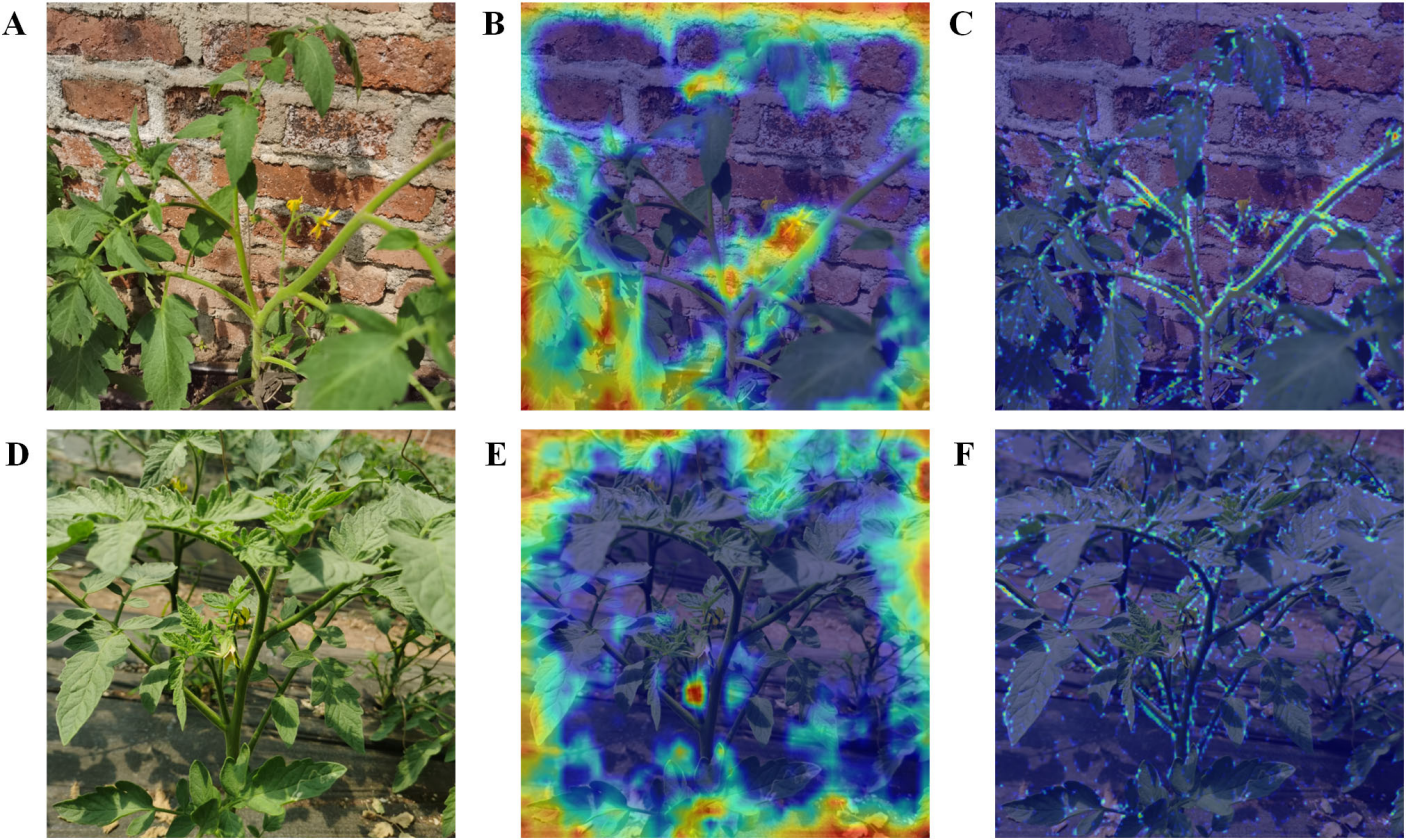
Grad-CAM-based weight heatmap. **(A)** Single background image. **(B)** YOLOv8n-seg. **(C)** YOLOv8n-seg+CBAM. **(D)** Complex background images. **(E)** YOLOv8n-seg. **(F)** YOLOv8n-seg+CBAM.

**Figure 10** shows weight heatmaps based on Grad-CAM. **Figures 10A**, **D** display the original images with simple and complex backgrounds, respectively. Visualizing the heatmaps for these two original images reveals **Figures 10B**, **E**. The YOLOv8n-seg model without attention mechanism shows scattered high-response areas. These mainly cluster around tomato stems and leaf edges, but it does not focus enough on key structural features of lateral shoots. Noticeable activation zones remain in background areas, indicating the model is still sensitive to irrelevant information during feature extraction. This lack of focus shows that the model fails to concentrate enough on target regions, leading to attention dispersion and feature interference. As a result, it has weak robustness in complex natural environments, causing frequent false positives and false negatives.

The improved model that incorporates the CBAM attention mechanism shows more focused and consistent high-response areas in the heatmaps of **Figures 10C**, **F** compared to the original model. These areas target the junctions between the main stem and lateral shoots of tomatoes, accurately identifying key structural features such as branch bifurcations and growth nodes. At the same time, background activation is significantly decreased. This indicates that the CBAM module effectively directs the network’s resource allocation during feature extraction through the combined effects of channel and spatial attention. As a result, the model can suppress irrelevant information while highlighting the features of target regions, improving its ability to recognize tomato lateral shoot structures and differentiate their characteristics.

The comparative results of Grad-CAM heatmaps clearly show the positive effect of the CBAM attention mechanism in improving the interpretability of the YOLOv8n-seg model. Adding this attention mechanism not only increases the model’s response strength to key areas but also enhances the focus of feature distribution. This qualitative interpretability analysis provides theoretical backing for future model refinement and optimization, while also giving intuitive visual evidence for intelligent recognition and decision-making in agricultural vision tasks.

## 5 Cutting decision mechanism

Although previous studies have improved lateral shoot detection accuracy, challenges remain in complex greenhouse environments. Occlusion, morphological ambiguity, and scale variation near the junction of lateral shoots and the main stem affect localization performance. These factors introduce uncertainty in cutting point estimation. Direct pruning based on a single detection result may cause missed shoots or unintended damage to the main stem. Therefore, uncertainty in cutting point localization should be explicitly modeled and quantified before pruning decisions are made. This provides reliability constraints for subsequent decision-making.

### 5.1 Uncertainty analysis of cutting points

#### 5.1.1 MC dropout-based cutting point sampling method

To characterize the uncertainty in lateral shoot cutting point localization, the MC Dropout strategy is introduced during the YOLOv8n-seg inference phase. Specifically, while keeping the model parameters unchanged, the Dropout layer within the network is activated and multiple random forward passes are performed to obtain a set of predictions with random perturbations.

For a single image undergoing *N* inferences, each lateral Shoot instance yields a set of cutting point coordinate distributions as defined in Equation 6.

(6) $$C_{i}=\{\left(x_{i}^{\left(r\right)},y_{i}^{\left(r\right)}\right)|r=1,2,\text{...},N\}$$

This set reflects the spatial distribution of cutting points under varying random perturbations. Where, denotes the cutting point coordinates obtained from the r-th inference, and N represents the number of inferences. In this study, *N* = 20.

#### 5.1.2 Cutting point extraction method and safety constraint strategy

For each lateral shoot segmentation mask obtained during inference, a morphological closing operation is applied to reduce noise. A skeletonization method is then used to extract the central structure of the lateral shoot. Connectivity of neighboring pixels in the skeleton image is analyzed to identify skeleton endpoints. The endpoint closest to the main stem is selected as the starting point of the lateral shoot.

**Figure 11:**
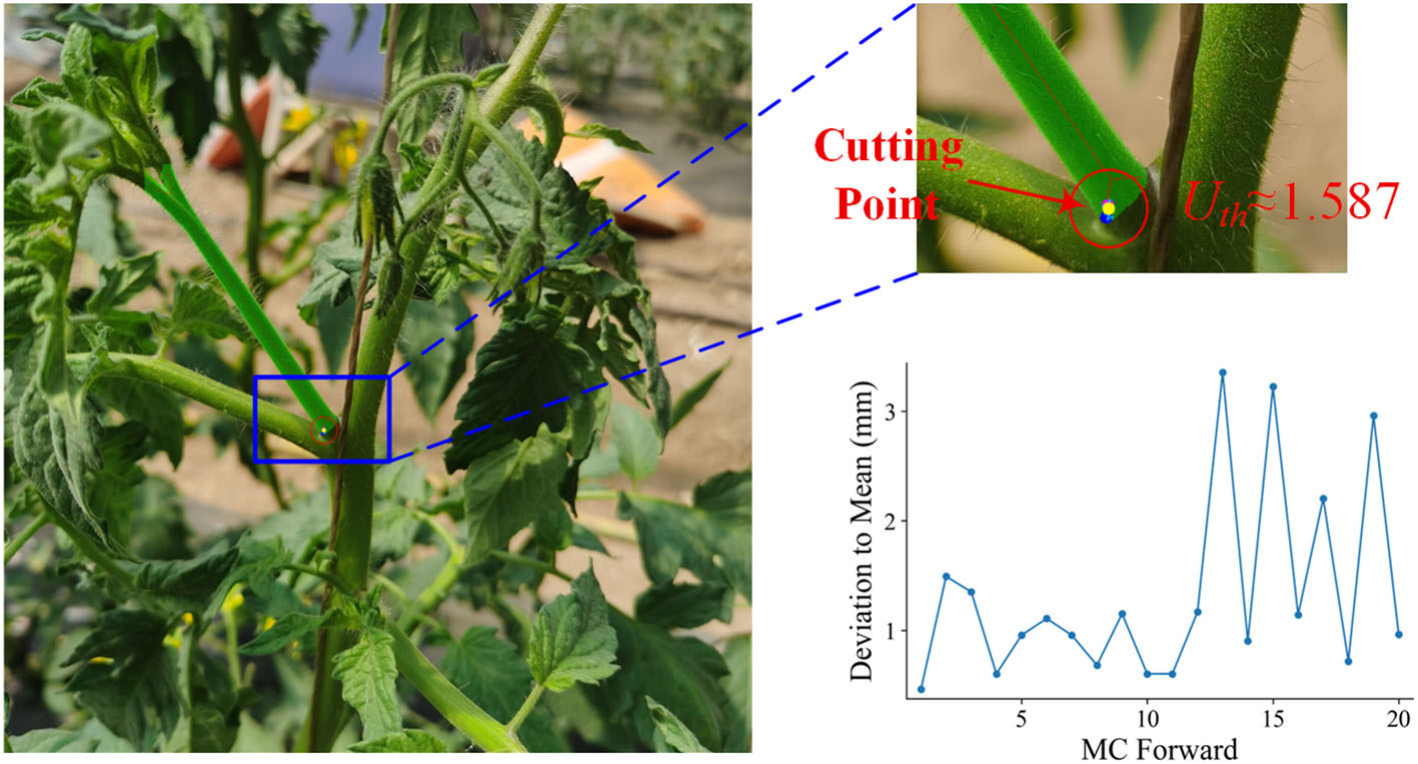
Uncertainty-aware cutting point extraction.

As shown in **Figure 11**, a minimum safety distance constraint is imposed on the skeleton to prevent cutting positions from being excessively close to the main stem. Candidate cutting points are generated along the skeleton structure under the safety constraint. Monte Carlo stochastic forward passes are further applied to obtain multiple cutting point samples for uncertainty analysis. The spatial distribution of these sampled cutting points is used to characterize prediction stability during pruning point estimation.

#### 5.1.3 Quantification method for cutting point uncertainty

Based on the results of multiple MC Dropout samples, the degree of spatial dispersion of the cutting points is used to quantify localization uncertainty.

The mean cutting point position is first computed as shown in Equation 7:

(7) $${\bar{x}}_{i}=\frac{1}{N}\sum_{r=1}^{N}x_{i}^{\left(r\right)},\bar{y}=\frac{1}{N}=\sum_{r=1}^{N}y_{i}^{\left(r\right)}$$

Indicates the estimated final position of the cut point.

The standard deviations in the horizontal and vertical directions are then calculated as shown in Equation 8:

(8) $$\sigma_{x}=\sqrt{\frac{1}{N}\sum_{r=1}^{N}{\left(x_{i}^{\left(r\right)}-{\bar{x}}_{i}\right)}^{2}},\sigma_{y}=\sqrt{\frac{1}{N}\sum_{r=1}^{N}{\left(y_{i}^{\left(r\right)}-{\bar{y}}_{i}\right)}^{2}}$$

Finally, the cutting point uncertainty is defined as shown in Equation 9:

(9) $$U_{i}=\frac{\sqrt{\sigma_{x}^{2}+\sigma_{y}^{2}}}{S}$$

where *S* represents the pixel-to-millimeter conversion factor, which is set to 4 pixels/mm in this study. This metric reflects the spatial dispersion of predicted cutting points. A larger value of *U_i_* indicates a higher positional uncertainty and a greater potential risk for pruning operations.

In this study, the uncertainty metric is used as a prediction stability indicator to characterize the consistency of cutting point estimation across stochastic forward passes.

### 5.2 LLM-assisted cutting decisions

By incorporating uncertainty analysis of pruning points, single-point pruning decisions are converted into decisions with confidence constraints. When uncertainty exceeds a preset threshold, automatic pruning is suppressed or manual review is triggered. This mechanism reduces the risk of incorrect pruning caused by unstable visual predictions in complex greenhouse environments.

However, uncertainty-constrained geometric analysis alone is still insufficient for practical pruning decisions. Whether tomato shoots require removal depends not only on geometric morphology but also on growth stage, spatial position, and pruning risk. Geometric information extracted by visual models cannot fully represent the implicit expertise and decision logic involved in agricultural practice. Therefore, an LLM is introduced to support high-level pruning decision-making based on structured perception features.

#### 5.2.1 Analysis of the limitations of rule-constrained pruning decision methods

In existing studies on automated tomato pruning, decisions are commonly based on manually defined constraints, such as shoot length thresholds, angle ranges relative to the main stem, and spatial relationships. These methods are applicable under simple structural conditions and stable environments. However, their performance is limited in practical greenhouse settings.

Tomato plants show notable morphological variation across growth stages and management conditions. The growth direction, thickness, and relative position of shoots change in a nonlinear manner. Single or limited rules cannot adequately represent diverse growth patterns. Under complex illumination and dense foliage shading, geometry-based rules that depend on detection results are affected by local misdetection and incomplete boundaries. This results in unstable pruning point estimation. In addition, rule-based methods have limited capacity to integrate contextual information. It is difficult to support robust decision-making that considers structural relationships among the main stem and shoots. Generalization ability is therefore constrained.

#### 5.2.2 LLM-assisted cutting decision framework

Relying only on fixed rules for pruning point inference does not satisfy the requirements of flexibility, adaptability, and robustness in greenhouse environments. Based on shoot identification and segmentation results, an LLM is introduced to support auxiliary pruning decisions. This module does not replace visual detection. It serves as a high-level semantic reasoning unit that analyzes the structured outputs of YOLOv8n-seg+CBAM.

In this study, we adopt Qwen-Plus, a large-scale language model provided by Alibaba Cloud, as the decision-making module. The model is accessed via the DashScope API with a fixed temperature setting (0.4) to ensure stable outputs.

**Figure 12:**
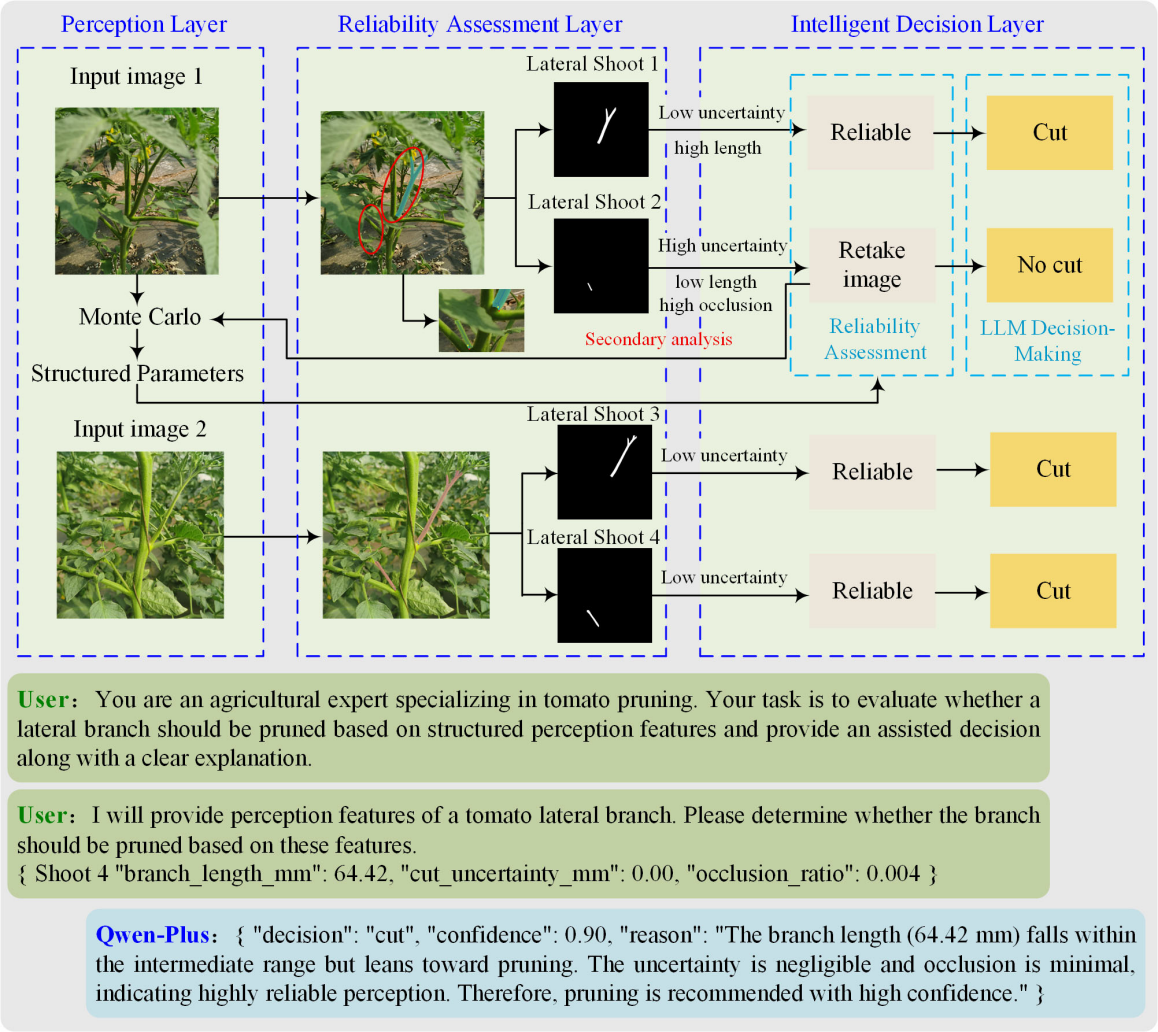
LLM-assisted pruning decision framework.

The overall framework of the proposed LLM-assisted pruning decision system is illustrated in **Figure 12**.

In the perception layer, the YOLOv8n-seg+CBAM model is used to extract structural information of tomato lateral shoots. Monte Carlo sampling is further employed to estimate the uncertainty of the predicted cutting point. Based on the segmentation results, a structured perception feature vector is constructed for each shoot, as defined in Equation 10:

(10) $$F_{i}^{p}=\left\{U_{i},L_{i},O_{i}\right\}$$

where *L_i_* denotes the branch length, *U_i_* represents the uncertainty of the cutting point estimation, and *O_i_* is the occlusion ratio.

The occlusion ratio *O_i_* is estimated based on the spatial compactness of the segmented shoot within its local region. It is defined in Equation 11:

(11) $$O_{i}=1-\frac{area(M_{i})}{area(bbox_{i})}$$

where *M_i_* denotes the segmentation mask of the *i*-th shoot, and bbox*_i_* represents the minimum enclosing bounding box of the corresponding mask.

The LLM is prompted to act as an agricultural expert and receives structured feature inputs in a predefined format. It outputs a pruning decision together with a confidence score and a reasoning explanation. A dialogue-based example of the prompt interaction is illustrated in **Figure 12**.

A representative case is shown for Lateral Shoot 4 in **Figure 12**. The shoot length of this shoot falls within the intermediate range (30–80 mm), where conventional rule-based methods typically adopt conservative strategies and defer the decision to manual judgment. According to the rule-based baseline, this shoot would not be automatically pruned.

However, the LLM produces a different decision. By jointly considering multiple perception features, including near-zero uncertainty and extremely low occlusion, the LLM determines that the perception result is highly reliable and that pruning can be safely executed. As a result, the LLM recommends a pruning action with high confidence.

This example highlights a key advantage of the proposed framework. While rule-based methods rely on rigid thresholds and often fail to handle ambiguous cases, the LLM is capable of integrating multi-dimensional information and performing contextual reasoning. In particular, for intermediate cases where traditional methods tend to be overly conservative, the LLM can leverage high-confidence perception to make more informed and proactive decisions.

The overall process of the cutting decision algorithm constructed by the aforementioned workflow is shown in **Algorithm 1**.

Algorithm 1

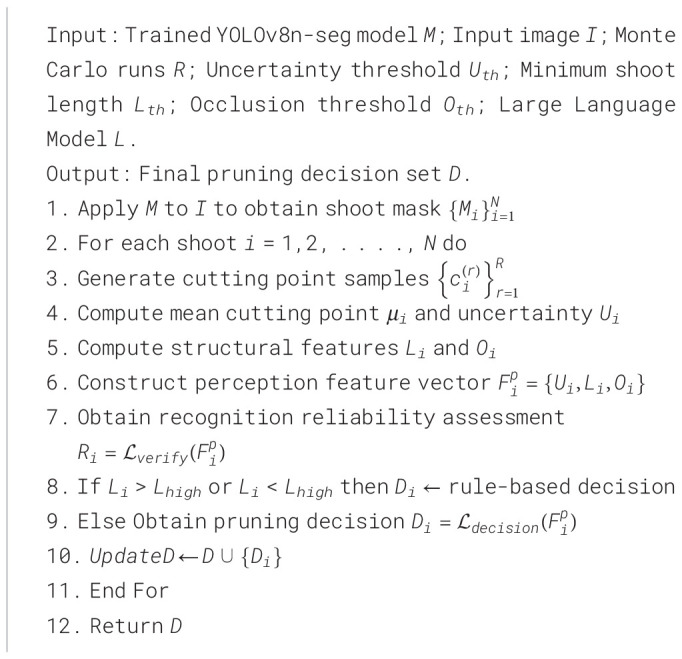

#### 5.2.3 Quantitative comparison between LLM-assisted and rule-based decisions

To quantitatively evaluate the proposed decision framework, a comparative experiment is conducted between the rule-based method and the LLM-assisted method using human expert annotations as the reference standard.

A total of 50 images are randomly selected from the test set, resulting in 81 lateral shoot instances. Each instance is evaluated by the rule-based method, the LLM-assisted method, and human experts.

The rule-based method determines pruning decisions solely based on branch length thresholds. Specifically, shoots longer than 80 mm are classified as “cut”, those shorter than 30 mm as “no cut”, while shoots within the intermediate range (30–80 mm) are conservatively assigned as “no cut” and deferred for manual inspection. In contrast, the LLM-assisted method follows the same rule constraints for clear cases, while intermediate cases are further evaluated using structured perception features.

The evaluation metrics include accuracy, precision, recall, and F1-score. These metrics are calculated based on standard classification definitions using the comparison between model predictions and human expert annotations. The F1-score is defined as the harmonic mean of precision and recall, which is calculated as shown in Equation 12:

(12) F1=2PrecisionRecallPrecicion+Recall

The results are presented in **Table 3**.

**Table 3:** Quantitative comparison between LLM-assisted and rule-based decisions.

Method	Accuracy/%	Precision/%	Recall/%	F_1_/%
Rule-based	72%	81%	48%	60%
LLM-assisted	76%	74%	78%	76%

The results show that the rule-based method achieves higher precision (*P* = 81%), indicating fewer incorrect pruning decisions. However, its recall (*R* = 48%) is relatively low, as many ambiguous cases are not actively resolved.

In contrast, the LLM-assisted method improves recall to 78% by making decisions in the intermediate range, while maintaining a comparable precision of 74%. As a result, the LLM-assisted method achieves a higher F1-score of 76%, compared to 60% for the rule-based method.

In addition, the proposed LLM-assisted framework effectively reduces the need for manual intervention. Under the rule-based strategy, 25 out of 81 samples (30.9%) fall within the intermediate range (30–80 mm) and are deferred for human inspection. In contrast, the LLM-assisted method actively resolves these ambiguous cases, thereby reducing the reliance on manual decision-making. This demonstrates the potential of the proposed approach to improve decision automation efficiency while maintaining reliable performance. The full pipeline processes 81 lateral shoot instances in 332.93s, corresponding to an average time of 4.11s per instance.

## 6 Conclusion

This study aims to automatically identify tomato lateral shoots in greenhouse environments and support intelligent pruning decisions. A dedicated tomato lateral shoot dataset was constructed. Considering challenges such as complex backgrounds, illumination variation, and difficulties in distinguishing shoot types, the YOLOv8 framework was investigated and improved by introducing an attention mechanism to enhance feature extraction while maintaining real-time detection performance. The main conclusions are summarized as follows.

1. To address environmental interference in greenhouse scenarios, including illumination variation, leaf occlusion, and small shoots, a diversified tomato shoot dataset was constructed and expanded through data augmentation. The experimental results demonstrate that the proposed framework maintains stable recognition performance under varying lighting conditions and complex backgrounds, showing good robustness for tomato lateral shoot perception tasks.
2. Several mainstream instance segmentation models were compared for agricultural applications with limited computational resources. The results show that YOLOv8n-seg achieves a favorable balance among segmentation accuracy, model size, and inference efficiency. After introducing the CBAM attention mechanism, the model further improves feature representation ability for small targets and complex backgrounds, leading to improved lateral shoot segmentation performance and enhanced interpretability.
3. An uncertainty quantification method combined with LLM-assisted decision making was proposed for tomato pruning scenarios. Monte Carlo sampling was used to estimate the uncertainty of cutting point localization, and structured perception features were integrated into the LLM-based decision framework. The proposed method enables pruning decisions with reliability constraints and improves decision consistency in ambiguous cases.

This study achieved a mAP_0.5_ of 97.7% using the YOLOv8n-seg+CBAM model on an NVIDIA RTX 4060 GPU, with a single-image inference time of approximately 8.0 ms. The hyperparameter configuration is listed in **Supplementary Table 4**. This demonstrates its applicability in agricultural scenarios by providing a visual perception solution for tomato lateral shoot identification and automated pruning systems. It also offers insights and methodologies for agricultural visual detection tasks in complex natural environments, as well as pruning tasks for grapevines and fruit tree shoots.

Although various data augmentation techniques were used to mimic different lighting conditions, the dataset was collected mainly from a single tomato cultivar under a controlled greenhouse environment. Therefore, performance fluctuations may still occur in practical applications involving different cultivars and growth conditions. Future work will further expand the diversity of the dataset to improve the robustness and generalization ability of the proposed framework across different environments. In addition, future studies will incorporate tomato lateral shoot cutting point localization and robotic arm control technology to enable automated pruning of tomato lateral shoots, promoting intelligent and precise agricultural production.

## Data Availability

The raw data supporting the conclusions of this article will be made available by the authors, without undue reservation.
