# Supplementary material for: Large language model assisted decision support framework for uncertainty aware detection and management of tomato lateral shoots
Source: Front Plant Sci. 2026 Jun 2;17:1853269. doi: 10.3389/fpls.2026.1853269 (PMC13269433; doi:10.3389/fpls.2026.1853269)
Supplement: Supplementary file 1 [file Table1.docx]

Supplementary Material

# Supplementary Figures and Tables

## Supplementary Figures


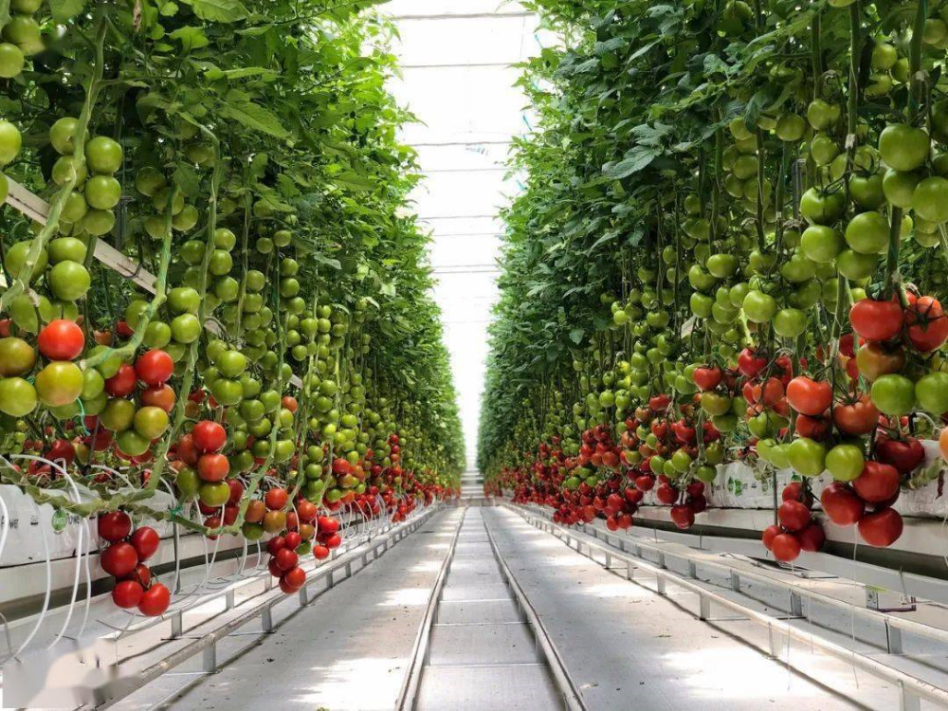


Supplementary Figure 1 Factory-grown Tomato Cultivation


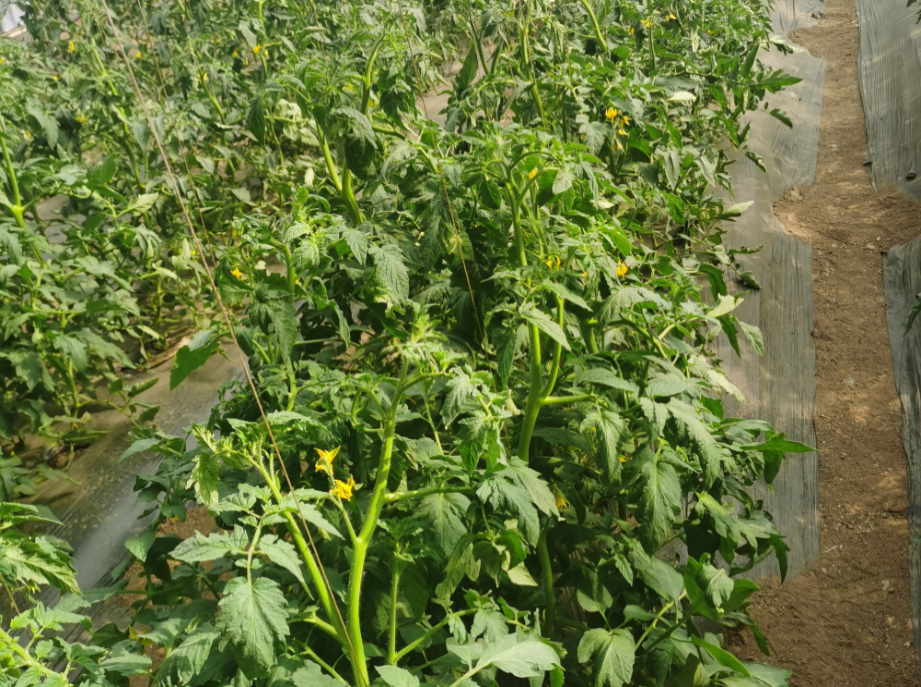


Supplementary Figure 2 Greenhouse Tomato Cultivation


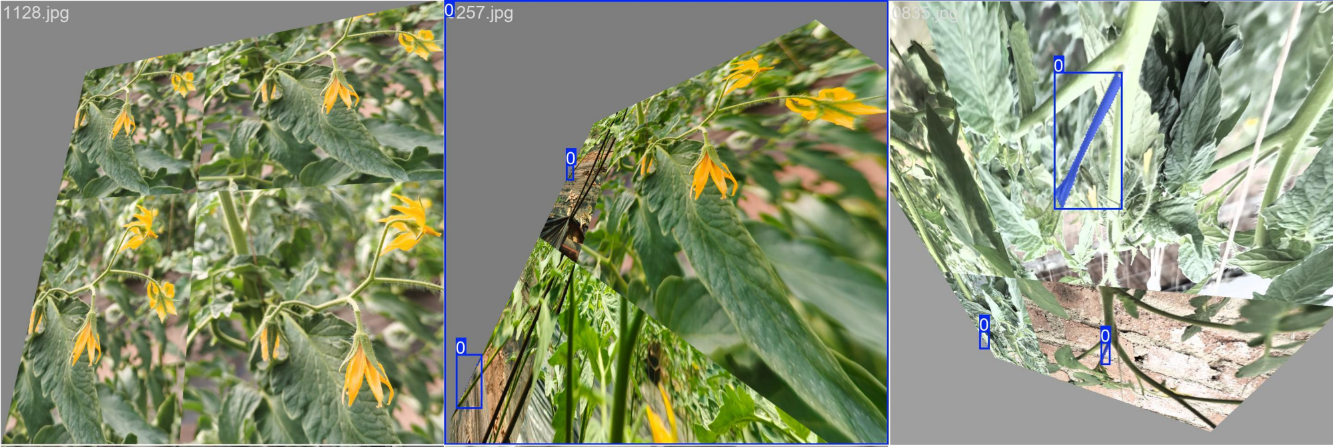


Supplementary Figure 3 The effect of data augmentation


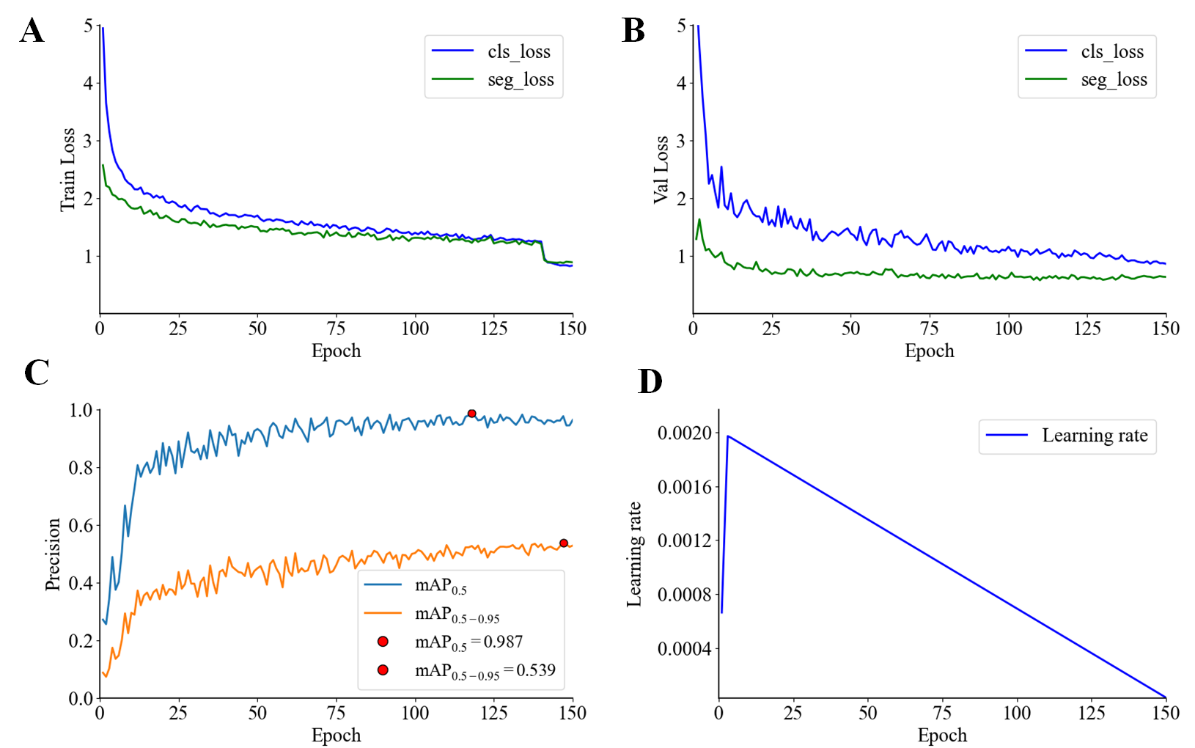


Supplementary Figure 4 YOLOv8n-seg Training Curve


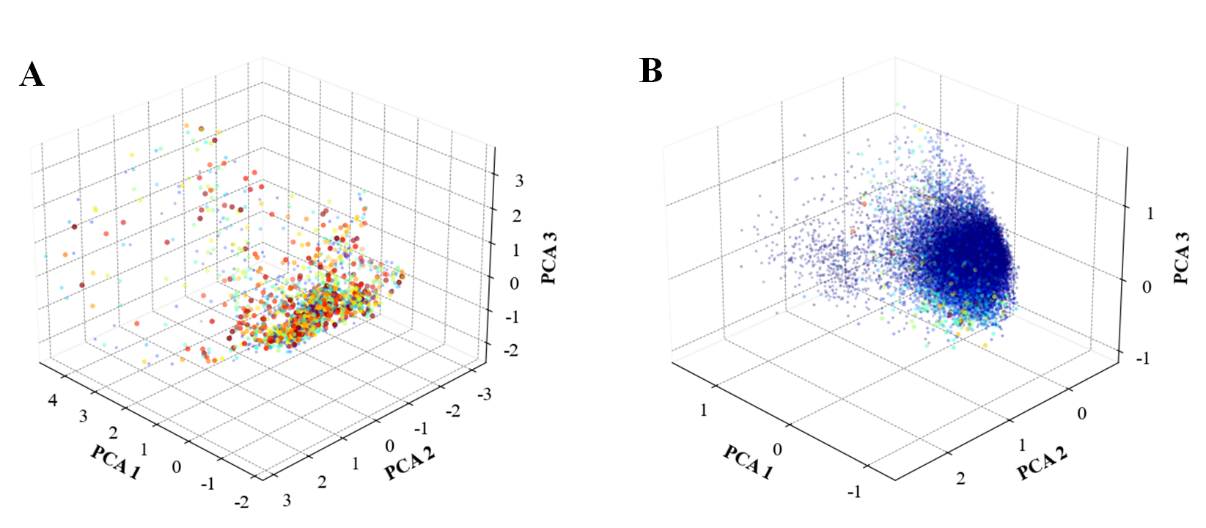


Supplementary Figure 5 The effect of data augmentation

## Supplementary Tables

Supplementary Table 1 Literature comparison

| References | Object Recognition | Basic Model | Improvement Strategy |
| --- | --- | --- | --- |
| Wang et al., 2023 | Tea leaves | Mask-RCNN | — |
| Nan et al., 2023 | Dragon fruit | YOLOv3 | Bottleneck、MetaAconC |
| Li et al., 2023 | Tea leaves | YOLOv4 | GhostNet、CBAM |
| Huang et al., 2025 | Citrus ripeness | YOLOv8 | ODConv、RepGFPN |
| He et al., 2025 | Estimating broccoli harvest readiness | YOLOv8n-seg | Triplet attention module |
| He et al., 2025 | Strawberry ripeness detection | YOLOv5s-cls | Stepwise Deep Learning |
| Peng et al., 2024 | Pest classification | MobileNet-V2 | PestNet |
| Dai et al., 2024 | Citrus diseases | YOLOv8 | Adown、BiFPN、GLSA |
| Chen et al., 2024 | Corn seed cracks | YOLOv8 | — |
| Kunduracioglu et al., 2024 | Grape leaf diseases | CNN | Digital image |
| Liu et al., 2024 | Green crisp plum | YOLOv8s-p2 | AFPN |
| Wang et al., 2023 | Tomato inspection | YOLOv5 | MobileNetV3-Large |
| Meng et al., 2024 | Tomato counting | YOLOv7 | SIMAM |
| Li et al., 2024 | Locating young mulberry leaves | YOLOv5 | CBAM |
| Liang et al., 2025 | Tomato pruning point | YOLOv5 | RepGFPN |
| Feng et al., 2022 | Pruning and defoliating tomato plants | Mask R-CNN | — |
| Liang et al., 2022 | Tomato side branch pruning point | Mask R-CNN | MobileNetv3-Large、ECA |
| Liang et al., 2025 | Nighttime tomato branch and leaves | YOLOv8 | CycleGAN |
| This study | Tomato lateral shoot | YOLOv8 | CBAM、LLM |

Supplementary Table 2 Data Augmentation Strategy and Parameter Configuration Table

| Augmentation Types | Configuration Items | Value | Description |
| --- | --- | --- | --- |
| HSV | hsv_h | 0.015 | Tone randomly adjusted within the range of [0, 0.015]. |
| HSV | hsv_s | 0.7 | Saturation randomly adjusted within the range of [0, 0.7]. |
| HSV | hsv_v | 0.4 | Random brightness adjustment, with values ranging from [0, 0.4] |
| Rotation | degrees | 0.3 | Random rotation angle, maximum rotation angle ±0.3 |
| Translation | translate | 0.1 | Random translation with up to 10% of the image size in displacement |
| Zoom | scale | 0.5 | Random scaling with a zoom ratio between 0.5x and 1.5x |
| Horizontal Flip | fliplr | 0.5 | Randomly flip the image horizontally with a 50% chance |
| Image Erasure | erasing | 0.4 | Randomly remove sections of the image with a 40% chance |
| Mosaic | mosaic | 1.0 | Randomly combine multiple images into a single image at 100% |
| Hybrid Enhancement | mixup | 0.2 | Using MixUp augmentation, the image and label blending ratio is 20% |
| Random Cropping | crop_fraction | 0.1 | Randomly crop the image while maintaining a 100% aspect ratio |
| Auto Enhance | auto_augment | randaugment | Using the RandAugment Automatic Enhancement Strategy |

Supplementary Table 3 Experimental Equipment Conditions

| Computer software and hardware | Version or Model |
| --- | --- |
| GPU | NVIDIA GeForce RTX 4060 |
| Python | 3.8.20 |
| CPU | i5-12490F |
| CUDA | 11.7 |

Supplementary Table 4 Hyperparameter Configuration Table

| Hyperparameter | Value |
| --- | --- |
| Input image size | 1280×1280 |
| Epochs | 150 |
| Batch size | 4 |
| Initial learning rate | 0.001 |
| Final learning rate factor | 0.01 |
| Weight decay | 0.001 |
